# Health Care Transformation Through Collaboration on Open-Source Informatics Projects: Integrating a Medical Applications Platform, Research Data Repository, and Patient Summarization

**DOI:** 10.2196/ijmr.2454

**Published:** 2013-05-30

**Authors:** Jeffrey G Klann, Allison B McCoy, Adam Wright, Nich Wattanasin, Dean F Sittig, Shawn N Murphy

**Affiliations:** ^1^Harvard Medical SchoolBoston, MAUnited States; ^2^Laboratory of Computer ScienceDepartment of MedicineMassachusetts General HospitalBoston, MAUnited States; ^3^Research ComputingInformation SystemsPartners Healthcare System, IncBoston, MAUnited States; ^4^The University of Texas School of Biomedical Informatics at HoustonHouston, TXUnited States; ^5^Department of General MedicineBrigham and Women's HospitalBoston, MAUnited States; ^6^Research ComputingInformation SystemsPartners Healthcare System, Inc.Boston, MAUnited States

**Keywords:** clinical information systems, medical informatics, knowledge bases, user-computer interface, data display, diffusion of innovation

## Abstract

**Background:**

The Strategic Health IT Advanced Research Projects (SHARP) program seeks to conquer well-understood challenges in medical informatics through breakthrough research. Two SHARP centers have found alignment in their methodological needs: (1) members of the National Center for Cognitive Informatics and Decision-making (NCCD) have developed knowledge bases to support problem-oriented summarizations of patient data, and (2) Substitutable Medical Apps, Reusable Technologies (SMART), which is a platform for reusable medical apps that can run on participating platforms connected to various electronic health records (EHR). Combining the work of these two centers will ensure wide dissemination of new methods for synthesized views of patient data. Informatics for Integrating Biology and the Bedside (i2b2) is an NIH-funded clinical research data repository platform in use at over 100 sites worldwide. By also working with a co-occurring initiative to SMART-enabling i2b2, we can confidently write one app that can be used extremely broadly.

**Objective:**

Our goal was to facilitate development of intuitive, problem-oriented views of the patient record using NCCD knowledge bases that would run in any EHR. To do this, we developed a collaboration between the two SHARPs and an NIH center, i2b2.

**Methods:**

First, we implemented collaborative tools to connect researchers at three institutions. Next, we developed a patient summarization app using the SMART platform and a previously validated NCCD problem-medication linkage knowledge base derived from the National Drug File-Reference Terminology (NDF-RT). Finally, to SMART-enable i2b2, we implemented two new Web service “cells” that expose the SMART application programming interface (API), and we made changes to the Web interface of i2b2 to host a “carousel” of SMART apps.

**Results:**

We deployed our SMART-based, NDF-RT-derived patient summarization app in this SMART-i2b2 container. It displays a problem-oriented view of medications and presents a line-graph display of laboratory results.

**Conclusions:**

This summarization app can be run in any EHR environment that either supports SMART or runs SMART-enabled i2b2. This i2b2 “clinical bridge” demonstrates a pathway for reusable app development that does not require EHR vendors to immediately adopt the SMART API. Apps can be developed in SMART and run by clinicians in the i2b2 repository, reusing clinical data extracted from EHRs. This may encourage the adoption of SMART by supporting SMART app development until EHRs adopt the platform. It also allows a new variety of clinical SMART apps, fueled by the broad aggregation of data types available in research repositories. The app (including its knowledge base) and SMART-i2b2 are open-source and freely available for download.

## Introduction

### Substitutable Medical Apps

The burden for development of innovative views of the medical record has, until recently, rested largely on the core software architects of electronic health record (EHR) systems. Local innovation on those systems has functionally been restricted to a small number of academic research hospitals with large research budgets [1], and their tools are frequently designed only for local use (eg, [2]). Transfer of local innovation to the larger medical community has often been slow and complex. For example, the WizOrder order-entry system, developed at Vanderbilt, is used widely within their hospital system and has been the source of much interesting research. However, WizOrder itself was unavailable to others until a commercial EHR vendor purchased it in 2001 [3], and it is now available only to users of that vendor system.

A new allocation of resources is emerging which will directly support distribution, modularity, and interoperability of local innovation. In 2009, Kohane and Mandl proposed that EHRs be designed as platforms for supporting modular third-party applications rather than as monolithic systems [4]. They drew analogies to “app stores” found in the smartphone market, where specialized applications are developed and purchased to meet niche or not-widely-understood needs, without compromising the basic integrity of the device. Such an ecosystem of apps, they suggested, would foster innovation without sacrificing compatibility. Users with particular information needs could become app developers and contribute innovative insights from their local environment to the larger medical informatics community.

In 2010, the United States Office of the National Coordinator for Health Information Technology (ONC) launched a four-year, $60 million government initiative: the Strategic Health IT Advanced Research Projects (SHARP) program. SHARP seeks to conquer well-understood challenges in medical informatics through breakthrough research. ONC funded four SHARP centers, one to study each of four challenge areas: information security, cognitive support, reusable applications, and secondary use of EHR data [5].

**Figure 1:**
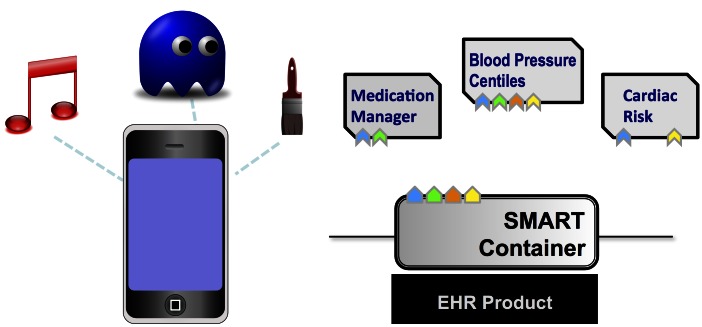
SMART enables an ecosystems of apps in medical systems, just as app stores enable this on smartphones. Portions adapted with permission from [4].

The Substitutable Medical Apps Reusable Technologies (SMART) center at Harvard Medical School is attempting to make Kohane and Mandl’s ecosystem for user-interface innovation a reality. SMART defines an application programming interface (API) and provides core software components so that health care information technology (HIT) systems’ developers can implement a SMART “container” interface to provide access to the data in EHRs in a standardized resource description framework (RDF) format. Apps written to conform to the container interface will run without modification on all EHRs and HIT systems that provide a SMART container. Apps can be written for patients, providers, and researchers, and all are backed by EHR data. The high level design is shown in Figure 1.

At the beginning of 2012, SMART leadership reported on their progress 14 months into the contract [6]. SMART had defined its initial API and had begun container development for three HIT platforms: an electronic health record system (OpenMRS), a personal health record system (Indivo), and a clinical research repository (Informatics for Integrating Biology and the Bedside, i2b2). They also developed a suite of charter apps. Most notably, SMART developers took a user-friendly conceptualization of a cardiac risk app that appeared in Wired magazine and converted it into a live SMART app in about a week [7]. SMART is extending its reach, recently implementing some of the SMART container interface on Cerner’s public API and developing an app to monitor trends in blood pressure and flag hypertension in pediatric patients [8]. This app has now been running at Children’s Hospital in Boston for several months and is seeing increased adoption each month. SMART hosted a national “app challenge”, which was won by HIT innovator Polyglot Systems for their “Meducation app”, providing multilingual, user-friendly medication instructions for patients [9,10]. A similar app challenge has just concluded for Indivo. The winner, Indivo WebNotes, allows users to integrate snippets from webpages directly into their personal health record [11]. Other SMART containers are also in development, including Mirth corporation’s work with SMART to enable two Health Information Exchanges [12]. SMART has also recently been supporting best practices in Continuity of Care Documents with a “report card” app that includes terminology validation and “soft” rubrics not included in the official validator [13].

### Patient Summarization

One potentially important use-case for SMART-style user-interface innovation is in clinical decision support (CDS). There is substantial evidence to suggest that CDS can be a powerful tool to improve the quality of patient care, yet commercial EHR systems have highly variable and underutilized CDS capabilities [14-16]. The Patient-Centered Cognitive Support SHARP, housed at the National Center for Cognitive Informatics and Decision Making (NCCD) in Houston, Texas, has the high-level goal of utilizing HIT to support clinician decision-making.

Its “automated model-based clinical summarization of key patient data” project seeks to make EHR data more easily digestible, particularly by transforming it from pages of disconnected data into a concise problem-oriented medical record (POMR). Clinical summarization is becoming particularly important given the overwhelming amount of information present in today’s EHRs. Sifting through these data present an added burden to already-overwhelmed clinicians, who admit to making mistakes due to hurry and distractions [17]. The POMR, first described by Weed in 1968, puts patients’ problems at the center of the record and organizes data around those problems [18]. Users have found this format facilitates quicker understanding and review, improved team communication, and faster auditing, among other advantages [19-23]. Although most commercial EHRs have some summarization capability, such summarizations focus on organizing each type of clinical data, rather than synthesized views of the patient record [24]. One evaluation study concluded that developing a suitable POMR “is not easy,” and that physicians have become accustomed to the standard time-oriented view [25], which suggests some of the reasons for sluggish change.

### SMART-i2b2

SMART’s app approach offers the ability to integrate a POMR view with traditional views of clinical data, and it also overcomes the difficulty of integrating new, vendor-independent applications with many vendor products. Therefore, we had previously turned to SMART and developed a proof-of-concept POMR SMART app [26]. However, SMART is not yet supported by many EHRs, which limited the utility of this line of development. In early 2012, we became aware of the SMART-i2b2 project. i2b2 is a flexible, componentized clinical data warehousing system that now enjoys widespread adoption as a research and population management data repository at over 100 sites worldwide. It is being developed as part of a National Institutes of Health (NIH)-funded center charged with developing a national computational infrastructure for biomedical computing [27]. SMART-enabling i2b2 has been underway for some time, but it has primarily focused on clinical research support, such as a patient-centric view for clinical trial recruitment selection [28].

**Figure 2:**
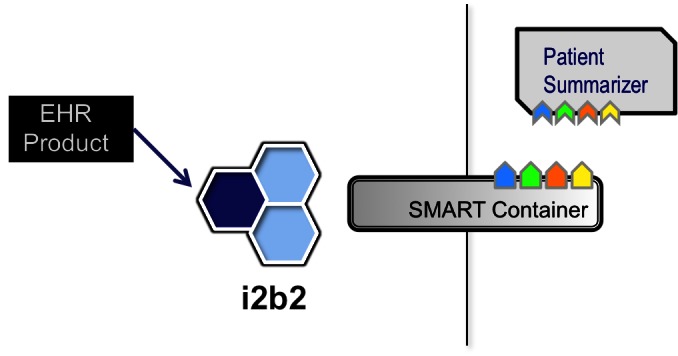
The overall architecture of the patient summarizer running in the SMART-i2b2 “clinical bridge”.

We theorized that i2b2’s popularity and the wealth of data available in i2b2 instances would make it a useful “clinical bridge”, to support SMART apps prior to large EHR vendors developing SMART containers. This led us to an architecture in which the patient summarization app runs in SMART-i2b2, shown in Figure 2. In this architecture, SMART-i2b2 would be launched as a webpage on the EHR workstation to run SMART clinical apps.

In this paper, we describe the results of our collaborative endeavor between i2b2 and the cognitive support and reusable apps SHARP centers, focused on creating more intuitive views of the EHR. Our goal was to facilitate development of intuitive, problem-oriented views of the patient record using NCCD knowledge bases that would run in any EHR.

## Methods

### Collaboration

To assist effective collaboration among sites, we used an Amazon virtual machine [29] to host our deployment of SMART-i2b2 with patient summarization, GitHub [30] to support collaborative development of the patient summarization app, DropBox [31] for sharing miscellaneous items such as notes and diagrams, and Google+ [32] to support multi-way real-time video conferencing. Tools like these will certainly become more important as collaborative, multi-site research is increasingly occurring [33].

### Patient Summarization

**Figure 3:**
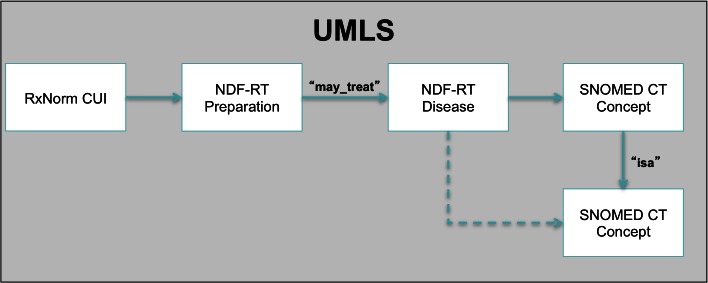
SMART RxNorm medications are mapped to SNOMED CT problems using the NDF-RT “may treat” linkage as intermediary. Adapted from [26]; used with permission.

The National Drug File Reference Terminology (NDF-RT) contains “may_treat” linkages between diagnoses and medications [34] which have been explored as a knowledge source for enhancing the problem list [35]. We have previously developed a proof-of-concept problem-medication linkage SMART app using an NDF-RT derived knowledge base [26]. For this work, we extended that app. Within the Unified Medical Language System (UMLS), there exist links between RxNorm (a SMART-approved terminology) and NDF-RT medication codes. From these medication codes, we traversed the “may_treat” linkages and then converted the linked NDF-RT diseases to the Systematized Nomenclature of Medicine-Clinical Terms (SNOMED-CT, another SMART-approved terminology) using the UMLS, expanding all levels of the problem hierarchy to create the most inclusive possible knowledge. The resulting knowledge base consists of over 7 million problem-medication links. An illustration of the knowledge base construction is shown in Figure 3. We stored our knowledge base as tuples of a related RxNorm medication concept unique identifier (CUI) and a SNOMED-CT problem code. To access this database from our Web application, we wrote a Web service in PHP Hypertext Preprocessor (PHP) to retrieve a list of SNOMED-CT problems given an RxNorm CUI. The NDF-RT approach is limited by knowledge gaps, in part due to data loss from incomplete mappings. However, this knowledge base is currently our most mature and it already uses SMART-required terminologies (SNOMED-CT and RxNorm). The SMART container handles mapping to these terminologies, so this knowledge base is “SMART ready.”

We are developing other knowledge bases using other techniques, each with strengths and weaknesses. One knowledge base utilizes probabilistic linkages in the medical record, an approach first suggested more than a decade ago [36]. This approach is able to detect correct linkage very accurately on medications used for one very specific purpose (eg, glycopyrrolate) and for non-clinical problems (eg, tube feeding, taking medication). As an example, our initial work with this method found the 50 strongest linkages in a dataset of 100,000 patients were all clinically accurate and the majority were for a very specific purpose [37]. This method does not require any effort by clinical experts, but the knowledge base must be recompiled in each setting, and it is less accurate on common diseases and interventions. Another knowledge base uses a form of crowdsourcing, which takes advantage of manually asserted links between problems and medications or laboratory results and is more accurate than probabilistic linkage on some multivariate associations, especially commonly prescribed medications with secondary problems (eg, metformin and polycystic ovarian syndrome [38-40]). While the crowdsourcing approach requires little effort to capture knowledge, methods must be applied to filter out noise (eg, patient data linked to a problem to facilitate billing and not medical care). A final approach utilizes a manually constructed knowledge base [41]. The manual approach had the highest accuracy but required the most effort and still only covered a relatively small number of common clinical conditions. Work has also been done on literature mining (such as PubMed and Food and Drug Administration product labels) to develop knowledge bases, though we have not yet incorporated this technique [42-45]. None of these knowledge bases have yet been sufficiently mapped to SMART-approved terminologies, so we used the NDF-RT approach for this work.

When the app is launched, it makes asynchronous JavaScript SMART API calls to retrieve demographics, medications, problems, allergies, lab results, encounters, vital signs, and immunizations. Each API call returns a SMARTResponse object containing a RDF graph containing that component of the patient record. For all objects except problems and medications, the app iteratively traverses each graph as it is retrieved to generate HTML display data. The app does not process problem and medication objects until both are loaded, because they must be handled together. When both are loaded, the app traverses the medication graph, retrieves all possibly related problems through the PHP Web service, and then traverses the problem graph, adding the current medication to the HTML output for all matching problems.

We modeled our summarization app’s user interface on a previously designed prototype interface of a problem-oriented view for OpenVista, which was evaluated using the Task, User, Representation, and Function framework for EHR usability [46,47]. We developed the app using HTML and JavaScript, facilitated by the Bootstrap front-end framework and the Google Visualization API [48]. The originally-developed proof-of-concept summarization SMART app showed all problems and medications on one screen, which can prove unwieldy for complex patients, and displayed output in a rigid HTML table. The new app, modeled after the prototype, features a responsive cascading style sheet, fluid grid design that ensures proper proportions for key screen resolutions and devices.

The app user interface displays the list of active problems on the left. Users may select a problem from the list to display associated medications on the right side. The user can also click the “All Medications” text to toggle a list of all prescribed medications for the patient. We have not yet integrated a knowledge base with lab results, so the app displays all historical lab results and vital signs in a list below the problems and medications. Users may click a lab result or vital sign to toggle display of the values; any lab result with multiple values is shown as a graph, generated using the Google Visualization API. See the Results section and Multimedia Appendix 1 for an example. The app is open-source and available for free download [30].

### SMART-i2b2

**Figure 4:**
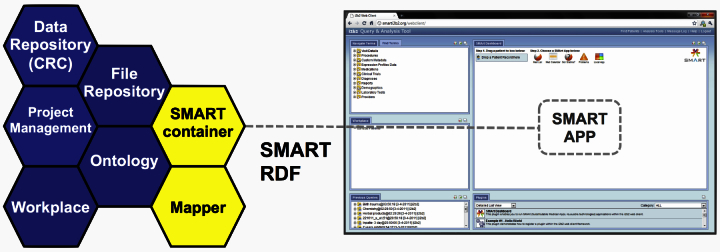
Left: i2b2 hive (blue) with SMART container and Mapper cells (yellow). Right: SMART Patient Centric view for the i2b2 Web interface.

i2b2 is a “hive” of “cells” (software modules), where each cell provides a set of Web services. New cells can be added to the hive and communicate with the other cells via Web service calls. The standard hive has the blue cells shown in Figure 4. Adding SMART functionality involved three changes to i2b2 [49].

First, we developed a new cell, the SMART container, which implements the SMART API and securely sends RDF messages to SMART apps as specified by the OAuth protocol. SMART places the burden of constructing valid SMART-RDF messages on the container developer. Therefore, we developed a flexible way in the SMART cell to transform an i2b2 XML message into a SMART-RDF XML message using stylesheets.

Second, in i2b2, one methodologically challenging piece is flexibly translating from the variety of i2b2 terminologies to the expected terminologies of SMART. To facilitate this translation, we developed a Mapper cell, which supports customizable mappings between terminologies and can be jumpstarted with existing linkages such as those in the UMLS. A set of about 2000 most used “target terms” for mapping, which covers 85% of terms used in the Partners Health care System [49,50], has been created and is distributed with the SMART-i2b2 container. These “target terms” are SNOMED-CT, RxNorm, and Logical Observation Identifier Names and Codes (LOINC) terms that can be loaded into the Mapper cell to provide guidance when an institution maps its local codes to the SMART preferred coding systems. Additionally, the i2b2 demonstration data’s terminology dictionary, which includes terms in the 9th edition of the International Classification of Diseases (ICD-9), NDF-RT, and several demographic value sets, was also mapped to SNOMED-CT and RxNorm. Although each i2b2 instance can choose which terminologies to support and therefore might require a custom mapping, many sites have adopted variations of the demonstration terminology.

The final change to i2b2 was an upgrade to the Web interface. The i2b2 Web interface supports plugins, and so a plugin was developed for the “SMART Patient Centric view”, shown on the right side of Figure 4. This EHR-like view in turn can be configured to run any number of SMART apps simultaneously, hosted locally and remotely. The Patient Centric view allows per-user organization of these apps into multiple views suited to the user’s needs.

With these aforementioned components, any SMART app can reside inside i2b2, communicating with i2b2 via the SMART container. These SMART-enabling components are freely available and can be installed as an add-on to any i2b2 installation [51].

## Results

We deployed i2b2 v1.6 in an Amazon-hosted virtual machine with the demonstration terminology dictionary and the 133 fake demonstration patients included in the standard release of i2b2. We then installed the SMART Web client plugin and SMART cells using the previously described “target mapping terms” list that was populated with mappings from this demonstration terminology dictionary. We were able to deploy our summarization app by adding it to the Web server hosting i2b2 and making a few small configuration changes.

**Figure 5:**
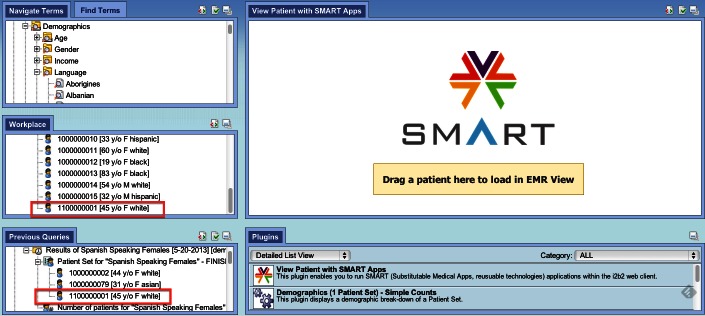
The i2b2 Web application with the SMART container activated. A patient can be dragged from a patient list in the workplace (first oval) or from a previous query result (second oval).

**Figure 6:**
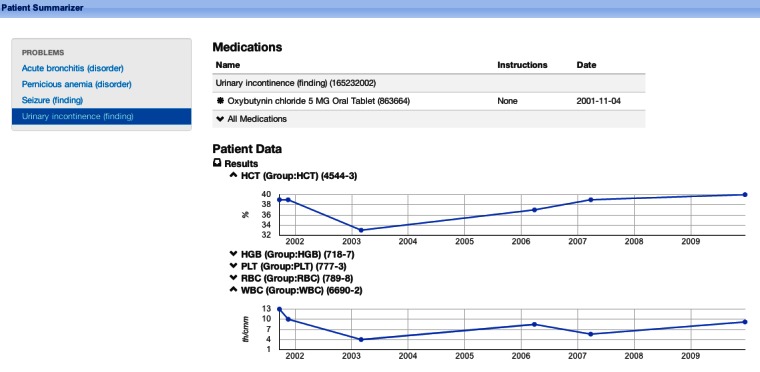
The patient summarization app running inside the SMART-i2b2 container. Shown here: urinary incontinence is highlighted and a relevant medication (oxybutynin) is displayed to the right; lab results are shown as line graphs below.

To run the app, a user chooses the SMART plugin inside the i2b2 Web client and drags the patient of interest into the Patient Centric view. One can either drag a patient from a customizable patient list (eg, the set of patients for which the user provides care), or from a previously executed research query. The two different options are shown in Figure 5. Once a patient is dragged into the Patient Centric view, the patient summarization app fills the screen (presuming the user has access to this patient’s data). The app is shown in Figure 6, and a demo is also included in Multimedia Appendix 1.

During development, we found that the engineered demonstration patients distributed with i2b2 tended to have uncorrelated problems and interventions, probably because they are not based on real patient data but only to meet the goal of testing research queries. Therefore, we developed a new test patient. Because we developed this test patient in i2b2 (using non-SMART terminologies like NDC and ICD-9), she was a patient who utilized the full translation pipeline from i2b2 to SMART, including the Mapper cell. Therefore, although she is still a test patient, we believe she comes close to a real-world i2b2 scenario, where local terms are dynamically mapped to SMART terminology. The app correctly found the problem-medication linkages shown in Table 1.

By SMART-enabling i2b2, we were able to develop a patient summarization app that can run in any i2b2 instance, reusing research data extracted from EHRs for clinical care. SMART-enabled i2b2 could then be launched as a webpage on an EHR workstation to run the summarization app on the current patient. We are finalizing a more streamlined workflow, in which the Patient Centric view can be launched for a particular patient separately from the full i2b2 Web client. This will allow easier access to clinical apps for a patient but still backed by the i2b2-SMART infrastructure.

**Table 1:** Problem-medication linkages found by the patient summarization app on our i2b2 test patient.

Problem	Medication
Acute bronchitis	Aminophylline 200 mg oral tablet
Pernicious anemia	Vitamin B12 1 mg/ml injectable solution
Seizure	Lamotrigine 100mg oral tablet
Urinary incontinence	Oxybutynin chloride 5 mg oral tablet

## Discussion

### Principal Findings

We successfully created a patient summarization app based on a validated NCCD knowledge base that can be run in nearly any EHR environment. For SMART-enabled EHRs, the app can be run directly within the EHR. In other cases, a SMART-enabled i2b2 instance can be used as a “sidecar” to the EHR, extracting data from it for research and clinical apps like this one. SMART-enabled i2b2 can run in a webpage alongside the EHR. This is certainly beneficial to more than 100 sites using i2b2 worldwide. We also found that online tools like Google+, GitHub, DropBox, and a cloud-hosted virtual machine increased our ability to collaborate effectively.

SMART has accelerated the implementation and testing of our patient summarization work. This step of our research was previously impeded by the need to maintain multiple apps for various clinical systems, thereby needing to adapt to each system’s local ontologies and local API. By choosing SMART, we can now test our knowledge bases using a single clinical app that will run in all SMART-enabled environments. At Partners’ Healthcare, this includes both i2b2 data repositories and directly in the outpatient medical record system. We are hopeful that supporting a single SMART app will allow us to disseminate our results both as a raw knowledge bases and as an executable tool.

Broad interest in SMART puts it in a good position to spread in the coming years, either through the demands and requirements of hospital systems, or through smaller EHR vendors implementing it in anticipation of gaining market share. Our hope is that this “sidecar” approach of running clinical apps through i2b2 will help foster SMART, by supporting SMART app development until EHRs adopt the platform. Furthermore, the i2b2 approach might provide SMART-specific functionality that are absent in other clinical systems.. Because i2b2 aggregates many systems’ data, it is able to provide more information than individual clinical systems, at the expense of real-time data. A SMART clinical app backed by i2b2 could allow clinicians to, for example, perform comparative effectiveness research on the fly to make treatment decisions for rare combinations of comorbidities [52].

Writing our SMART app was not particularly time-consuming; the majority of the work was developing the SMART-i2b2 container and NCCD knowledge bases. This indicates that SMART might be an ideal platform for quick dissemination of innovative tools. It is also notable that SMART apps will naturally become easier to write as general Internet innovation flourishes, because SMART apps can leverage freely available Web development toolkits such as Bootstrap and the Google Visualization API. While only about a dozen SMART apps have been developed to date, SMART has already enabled small software shops to innovate on EHR data through the SMART “app challenges”.

Whether SMART becomes the de facto standard for EHR apps remains to be seen as the platform matures. Already it has several points in its favor. First, it lessens the learning curve of app development by leveraging existing Web standards (eg, JavaScript Object Notation data structures and Web service interfaces). Second, the current API is a straightforward RDF data model designed to meet the needs of app development without trying to solve all use-cases for external views of clinical data. This avoids the steep learning curve of formats such as the Clinical Document Architecture, a health care data standard used for representing all types of clinical data. Third, SMART’s current read-only approach will be extended in the future with methods to write data back to the record. SMART enables clinical app innovation by giving app developers access to clinical data elements on individual patients, and it is complemented by data analytical platforms such as i2b2 (for aggregate, research-oriented data repositories and reporting).

### Challenges

The greatest challenges we faced in this endeavor occurred in “gluing” the pieces together. The downloadable source code [51], which was in its early stages during development, did not include usable default configuration files and provided scant documentation. However, this has since been resolved. Some user-interface changes were necessary in the app. For example, the SMART-i2b2 container provides a panel of demographic information that the “SMART sandbox” implementation does not. We also modified the app to only display one instance of a problem, because i2b2 returns all historical diagnoses of that problem. We further hid the allergies and vitals sections, which were not supported in SMART-i2b2 when we deployed the app. As discussed, we discovered the developer-engineered i2b2 sample patients were not suitable for problem-oriented analysis, which required that we develop our own. Finally, the SMART API changed several times during development, requiring frequent minor changes to the app. All these issues were associated with platform development, and are not expected to recur.

As the technology matures, installing and developing containers and deploying apps will become simpler. The longer-term challenge for SMART deployments will be terminology mappings. This is a barrier to interoperability in general, and it appears in almost all health information exchange problems in medical informatics—from generating conformant continuity of care documents to consuming quality measure queries. Advanced methods for mapping terminologies are necessary. The i2b2 platform utilizes a mapping tool that extracts terms from the National Center for Biomedical Ontology. This is freely available and has been integrated into the SMART-i2b2 platform [53]. Drawing from tools like these and those provided by the UMLS will be a good starting point, but it is possible that other methods, such as crowdsourcing or probabilistic linkage, will become important in terminology mapping as well.

### Future Plans

Previous testing of ontology-based knowledge bases on real patient data showed poor sensitivity [26], which could be partially attributed to information loss during mapping. Once mapped to SMART-approved terminologies, our other knowledge bases (those developed through crowdsourcing, probabilistic linkage, and expert design) could be integrated into the summarization app. We suspect that by combining these knowledge bases (eg, by joining them or with a probabilistic-weighting approach) the coverage of our app will far exceed what we have demonstrated here. At that point, a new evaluation using real patient data would be appropriate.

Also, although we have extended the SMART app beyond the original prototype, it does not yet have the full functionality of the usability-tested prototype interface, nor does it currently have a particularly compelling “look and feel”. Beyond further refinement of the user interface, the app will need improvements of its handling of SMART patient data. Our current app simply displays the most recent problem and medication instance rather than a summary of that problem or medication’s history. Also, methods should be developed to incorporate unencoded data into the summary, as SMART does not require every problem or medication to have an associated code.

For the SMART platform as a whole, there are several open questions. One is the level of programming expertise needed to build an app. SMART supports app distribution in an interoperable environment and it lowers the entry threshold for those interested in developing innovative apps. However, we have not yet evaluated what average EHR users can accomplish with SMART. Currently available SMART apps have been written by groups with significant prior programming experience. A second open question is the appropriate distribution model for these apps. The iPhone app store has a certification process, whereas the Android app store does not. Because SMART’s goal is to foster innovation, it does not seem wise to restrict distribution of apps. Instead, some type of certification for apps performing key clinical functions might be needed. Currently, the ONC’s certification criteria for EHR systems require that any component performing a function for which certification exists must be certified for that function [54]. One approach moving forward might include ONC certification of SMART apps through similar testing mechanisms. However, stringing together many certified technological components does not necessarily mean that the entire system would perform correctly. For example, even if i2b2, its SMART container, and a patient summarization app were all somehow certified, an improper deployment or poor mappings could still cause the app to miss important information in its synthesis. This is a challenging problem that might require more complicated certification criteria.

### Conclusions

We have successfully deployed a patient summarization app in the i2b2 clinical data repository platform. This provides a problem-oriented view of the medical record by combining a previously developed knowledge base and the SMART medical apps platform. It leverages co-occurring work in building a SMART-i2b2 container for research, so that this clinical app can be available to the many clinicians whose information systems include i2b2 but do not otherwise have access to SMART. This technical work lays the foundation for a broader ecosystem of reusable apps to provide innovative summary views of the health record. It also provides a “clinical bridge” an i2b2-based pathway for reusable app development that does not require EHR vendors to immediately adopt the SMART API. We hope this will support SMART app development until EHRs adopt the platform.

All software components discussed here are freely available for download, including i2b2, SMART, the SMART-i2b2 integration, and the patient summarization app [30,51].

## Multimedia Appendix 1

Screencast demo of patient summarization app running inside SMART-i2b2. This is a primarily a technical demonstration of the system, and the patient shown in the example does not necessarily have comorbidities that are realistic or clinically interesting.

## Abbreviations

API: application programming interface
CDS: clinical decision support
CSS: cascading style sheet
CUI: concept unique identifier
EHR: electronic health records
HIT: health care information technology
HTML: hypertext markup language
i2b2: Informatics for Integrating Biology and the Bedside
ICD-9: International Classification of Diseases, 9th edition
LOINC: Logical Observation Identifier Names and Codes
NCBO: National Center for Biomedical Ontology
NCCD: National Center for Cognitive Informatics and Decision-making
NDF-RT: National Drug File - Reference Terminology
NIH: National Institutes of Health
ONC: Office of the National Coordinator for Health Information Technology
PHP: PHP Hypertext Preprocessor
POMR: problem-oriented medical record
RDF: resource description framework
SHARP: Strategic Health Information Technology Advanced Research Projects
SMART: Substitutable Medical Apps, Reusable Technologies
SNOMED-CT: Systematized Nomenclature of Medicine-Clinical Terms
UMLS: Unified Medical Language System
